# The Role of Antibiotics in Endoscopic Transmural Drainage of Post-Inflammatory Pancreatic and Peripancreatic Fluid Collections

**DOI:** 10.3389/fcimb.2022.939138

**Published:** 2022-07-05

**Authors:** Mateusz Jagielski, Wojciech Kupczyk, Jacek Piątkowski, Marek Jackowski

**Affiliations:** Department of General, Gastroenterological and Oncological Surgery, Collegium Medicum Nicolaus Copernicus University, Toruń, Poland

**Keywords:** antibiotics, antibiotic therapy, pancreatic fluid collection, pancreatitis, antibiotic prophylaxis, endoscopic drainage, endoscopy

## Abstract

**Background:**

Although endoscopic treatment of symptomatic post-inflammatory pancreatic and peripancreatic fluid collections (PPPFCs) is an established treatment method, some aspects of endotherapy and periprocedural management remain controversial. The role of antibiotics is one of the most controversial issues in interventional endoscopic management of local complications of pancreatitis.

**Methods:**

This study was a randomized, non-inferiority, placebo-controlled, and double-blinded clinical trial to investigate the role of antibiotic prophylaxis in endoscopic transmural drainage in patients with symptomatic non-infected PPPFCs and assess the influence of antibiotic treatment on the results of endotherapy in patients with symptomatic infected PPPFCs.

This trial included 62 patients treated endoscopically for PPPFCs in 2020 at our medical center. Patients were divided into two groups; group 1 comprised patients who had received empirical intravenous antibiotic therapy during endotherapy and group 2 comprised patients who did not receive antibiotic therapy during endoscopic drainage of PPPFCs. The end points were clinical success and long-term success of endoscopic treatment.

**Results:**

Thirty-one patients were included in group 1 (walled-off pancreatic necrosis [WOPN, 51.6%; pseudocyst, 48.4%) and 31 patients in group 2 (WOPN, 58.1%; pseudocyst, 41.9%) (p=0.6098/nonsignificant statistical [NS]). Infection with PPPFCs was observed in 15/31 (48.39%) patients in group 1 and in 15/31 (48.39%) patients in group 2 (p=1.0/NS). The average time of active (with flushing through nasocystic drainage) drainage in group 1 was 13.0 (6 – 21) days and was 14.0 (7 – 25) days in group 2 (p=0.405/NS). The average total number endoscopic procedures on one patient was 3.3 (2 – 5) in group 1 and 3.4 (2 – 7) in group 2 (p=0.899/NS). Clinical success of PPPFCs was observed in 29/31 (93.5%) patients from group 1 and in 30/31 (96.8%) patients from group 2 (p=0.5540/NS). Complications of endotherapy were noted in 8/31 (25.8%) patients in group 1 and in 10/31 (32.3%) patients in group 2 (p=0.576/NS). Long-term success in group 1 and 2 was reported in 26/31 (83.9%) and 24/31 (77.4%) patients, respectively (p=0.520/NS).

**Conclusions:**

The effective endoscopic drainage of sterile PPPFCs requires no preventive or prophylactic use of antibiotics. In infected PPPFCs, antibiotic therapy is not required for effective endoscopic transmural drainage.

## Introduction

The history of pancreatitis may involve the development of four types of post-inflammatory pancreatic and peripancreatic fluid collections (PPPFCs) as local complications of acute inflammation, including acute peripancreatic fluid collection, pancreatic pseudocysts, acute necrotic collection, and walled-off pancreatic necrosis (WOPN) (Thoeni, 2012; Banks et al., 2013; Sarr et al., 2013). Each collection may be sterile (non-infected) or infected (Thoeni, 2012; Banks et al., 2013; Sarr et al., 2013; Sarathi Patra et al., 2014; Manrai et al., 2018). For many years, the traditional management of local complications of acute pancreatitis consisted of surgical treatment combined with intravenous antibiotic therapy, particularly in cases of suspected tissue infection (Loveday et al., 2008; da Costa et al., 2014). In recent decades, advances have been made in minimally invasive methods for the treatment of post-inflammatory PPFCs, such as endoscopic techniques, which lead to radically shortened recovery times and lower complication and mortality rates (Loveday et al., 2008; Freeman et al., 2012; da Costa et al., 2014; Szeliga and Jackowski, 2014; Jagielski et al., 2018; Jagielski et al., 2020).

Endoscopic transmural drainage involves creating a fistula between the PPPFC cavity and the lumen of the gastrointestinal tract to facilitate free drainage of the collection contents into the digestive tract (Loveday et al., 2008; Freeman et al., 2012; da Costa et al., 2014; Jagielski et al., 2018; Jagielski et al., 2020). During endoscopic transmural drainage of post-inflammatory PPFCs, transmural puncture of the PPPFC is performed under endoscopic ultrasound (EUS) guidance (Loveday et al., 2008; Freeman et al., 2012; da Costa et al., 2014; Jagielski et al., 2018; Jagielski et al., 2020). Next, the puncture site was dilated using a cystostome to form a transmural fistula, a connection between the upper gastrointestinal tract (stomach or duodenum) and PPPFC (Loveday et al., 2008; Freeman et al., 2012; Jagielski et al., 2018; Jagielski et al., 2020). The next stage of the endoscopic procedure consists of enlarging the pancreatogastric or pancreatoduodenal fistula (Loveday et al., 2008; Freeman et al., 2012; da Costa et al., 2014; Jagielski et al., 2018; Jagielski et al., 2020). After enlargement, a self-expanding stent (lumen-apposing metal stent [LAMS]) or plastic endoprosthesis(-ses) is introduced *via* the fistula to enable free passive transmural drainage of the collection contents into the lumen of the gastrointestinal tract (Arvanitakis et al., 2018; Jagielski et al., 2018; Jagielski et al., 2020; Jagielski and Jackowski, 2021). Passive transmural drainage (without flushing through nasocystic drainage) is an effective method for endoscopic treatment of sterile pancreatic pseudocysts with liquid serous content alone (Arvanitakis et al., 2018; Jagielski et al., 2018; Jagielski et al., 2020; Jagielski and Jackowski, 2021). With regards to infected pseudocysts and sterile or infected necrotic collections that contain necrotic tissue in addition to liquefied necrotic contents, active (with flushing through nasocystic drainage) transmural drainage is required, consisting of additional saline irrigation introduced *via* the fistula to rinse the collection cavity in the postoperative period (Arvanitakis et al., 2018; Jagielski et al., 2018; Jagielski et al., 2020; Jagielski and Jackowski, 2021).

Although endoscopic treatment of symptomatic PPPFCs due to pancreatitis is an established treatment method, some aspects of endotherapy and periprocedural management remain contentious (Arvanitakis et al., 2018; Jagielski et al., 2018; Jagielski et al., 2020; Jagielski and Jackowski, 2021);. The role of antibiotics is one of the most controversial issues in interventional endoscopic management of local complications of pancreatitis.

Antibiotic therapy is an important element in the conservative treatment of acute pancreatitis (Tenner et al., 2013; Working Group IAP/APA, 2013; Arvanitakis et al., 2018). The primary indication for the initiation of antibiotic therapy in patients with acute pancreatitis is confirmed pancreatic or extrapancreatic infection (Tenner et al., 2013; Working Group IAP/APA, 2013; Arvanitakis et al., 2018); other indications remain unclear. Prophylactic antibiotics, to prevent infection of necrotic areas, are not recommended in patients with acute pancreatitis (Tenner et al., 2013; Working Group IAP/APA, 2013; Arvanitakis et al., 2018; Crockett et al., 2018) and their overall use in acute pancreatitis remains controversial despite numerous publications. Furthermore, the optimum duration of antibiotic therapy in acute pancreatitis is unknown.

Similar controversies have been raised regarding the use of antibiotics in interventional gastrointestinal endoscopy, which remain the subject of numerous studies. According to available guidelines, the use of antibiotic prophylaxis for gastrointestinal endoscopy should be determined mainly by risk evaluation of bacteremia associated with endoscopic procedures (ASGE Standards of Practice Committee, 2015). Bacterial translocation of endogenous microbial flora into the bloodstream (bacteremia) may occur during endoscopy as consequence of gastrointestinal wall’s trauma related to the procedure (ASGE Standards of Practice Committee, 2015). The highest rates of bacteremia have been reported with esophageal dilation, sclerotherapy of varices in the upper gastrointestinal tract, percutaneous endoscopic feeding tube placement and endoscopic instrumentation of obstructed bile ducts (ASGE Standards of Practice Committee, 2015). In these cases antibiotic prophylaxis is indicated (ASGE Standards of Practice Committee, 2015). In case of EUS-guided fine-needle aspiration (EUS-FNA) the use of antibiotic prophylaxis depends on type of punctured lesion (Barkay et al., 2009; ASGE Standards of Practice Committee, 2015; Polkowski et al., 2017; Facciorusso et al., 2019; Colán-Hernández et al., 2020). Prophylactic antibiotics are not recommended prior to EUS-FNA of solid lesions (ASGE Standards of Practice Committee, 2015; Polkowski et al., 2017). On the other hand, administration of antibiotics has been recommended before EUS-FNA of cystic lesions (Barkay et al., 2009; ASGE Standards of Practice Committee, 2015; Polkowski et al., 2017; Facciorusso et al., 2019; Colán-Hernández et al., 2020). It is also worth to pay attention to use of antibiotic prophylaxis during endoscopic retrograde cholangiopancreatography (ERCP). It is recommended in patients with bile duct obstruction in absence of cholangitis during ERCP with incomplete biliary drainage in order to prevention of cholangitis (ASGE Standards of Practice Committee, 2015). In case of patients with bile duct obstruction in absence of cholangitis during ERCP with complete biliary drainage antibiotic prophylaxis is not required (ASGE Standards of Practice Committee, 2015). Our study based on similar assumption: effective (complete) endoscopic drainage of PPPFCs does not require antibiotic prophylaxis in order to prevention of infection or superinfection of PPPFCs.

The importance of prophylactic antibiotics in invasive endoscopic procedures in the pancreatic field remains unknown. Currently, no clear guidelines are available regarding the need for periprocedural antibiotic prophylaxis or its duration. This study attempted to define the role of antibiotics in the endoscopic treatment of PPPFC.

## Materials and Methods

This study was conducted in accordance with the Consolidated Standards of Reporting Trials (CONSORT) (Turner et al., 2012). This study was a randomized, non-inferiority, placebo-controlled, and double-blinded clinical trial to investigate the role of antibiotic prophylaxis in endoscopic transmural drainage in patients with symptomatic non-infected PPPFCs and assess the influence of antibiotic treatment on the results of endotherapy in patients with symptomatic infected PPPFCs.

It has been hypothesized that the efficiency of endoscopic PPPFC drainage is the basic criterion for therapeutic success, regardless of the infectious agent in PPPFC. The results of endoscopic treatment were based on effective drainage, regardless of antibiotic prophylaxis, and whether antibiotic therapy was used. This hypothesis was verified by examining the effect of antibiotic prophylaxis in patients with sterile (non-infected) PPPFC and antibiotic therapy in patients with infected PPPFC on the efficacy and safety of endoscopic transmural drainage.

Herein, the primary objective was to investigate the influence of antibiotic prophylaxis on the efficiency and safety of endoscopic transmural drainage in patients with sterile (non-infected) PPPFCs and antibiotic treatment in patients with infected PPPFCs.

The secondary objectives were to assess the influence of antibiotic prophylaxis and treatment on the duration of endotherapy and the number of endoscopic procedures (aggressiveness of endotherapy) in patients with non-infected and infected PPPFCs.

This study was conducted at the Department of General, Gastroenterological, and Oncological Surgery, Ludwik Rydygier Collegium Medicum in Bydgoszcz, Nicolaus Copernicus University in Toruń in 2020.

The study was approved by the Ethics Committee at the Collegium Medicum of Nicolaus Copernicus University (Approval Number KB 294/2020) and was conducted in accordance with the Declaration of Helsinki. All patients provided oral and written informed consent before inclusion in the study. All patients received detailed information regarding the study.

The recruitment period was from 01/01/2020 to 31/12/2020. The observation lasted 12 months from the end of endotherapy (until December 31, 2021). The entire study period was from 01/01/2020 to 31/12/2021.

The diagnosis of pancreatitis, criteria of clinical and morphological categorization, and all definitions were based on the 2012 revised Atlanta classification (Thoeni, 2012; Banks et al., 2013; Sarr et al., 2013). The standards for conservative treatment of pancreatitis are based on commonly available international guidelines (Tenner et al., 2013; Working Group IAP/APA, 2013);. Additional treatment methods were used depending on the concomitant organ impairment and the patient’s overall clinical condition. Each individual case of a patient with pancreatitis was thoroughly discussed during interdisciplinary meetings of senior staff. Decisions were made regarding further management of the patient and the potential rationale for interventional treatment.

### Study Inclusion Criteria

All consecutive patients with symptomatic post-inflammatory PPFCs in the late stage of pancreatitis (> four weeks from the onset of the disease) were included in this study. All patients aged 18 years were included in this study. All patients with clinical symptoms related to PPPFCs due to acute or chronic pancreatitis were enrolled. All patients who underwent endoscopic drainage for symptomatic PPPFCs in the late stage of pancreatitis were also included. Qualification for endoscopic intervention was based on clinical picture and imaging results, primarily abdominal contrast-enhanced computed tomography (CECT).

All patients were clinically assessed using the Sequential Organ Failure Assessment (SOFA) score/quick Sequential Organ Failure Assessment (qSOFA) score (Vincent et al., 1996; Vincent et al., 1998; Barbara et al., 2018).

Patients were included irrespective of existing suspicion of infection of collection’s content (both patients with infected PPPFCs and with sterile/non-infected PPPFCs) if the dynamics of change in SOFA and qSOFA scores during the endoscopic treatment did not exceed two (≤2) points in infected PPPFCs.

### Study Exclusion Criteria

Patients aged < 18 years and pregnant women were excluded from the study. Patients with PPPFCs that were not a consequence of pancreatic inflammatory diseases were excluded from the study (1 patient). Patients with post-inflammatory PPFCs without clinical symptoms were excluded (24 patients). Patients who had undergone interventional endoscopic treatment in the early phase of pancreatitis (< four weeks from disease onset) were also excluded (6 patients). Infected PPPFCs patients with SOFA/qSOFA scores that exceeded two (>2) points were excluded from the study (13 patients).

Additional exclusion criteria were as follows: antibiotic therapy for any other indication 7 days before the endoscopic procedure (11 patients) and allergy to antibiotics (piperacillin or tazobactam) (1 patient).

After meeting the exclusion criteria, 56 patients were excluded from the study.

### Study Group

After meeting the inclusion and exclusion criteria, the study group consisted of patients who underwent endoscopic transmural drainage of symptomatic post-inflammatory PPFCs. Patients in the study group were randomly assigned to the antibiotic group (group 1) or placebo group (group 2). The mechanism of random allocation of patients to each group was based on the randomness of the allocation resulting from the order of clinically necessary endotherapeutic interventions. Simple randomization was used, assigning consecutive patients undergoing endoscopic treatment to group 1 or group 2. Allocation to the antibiotic or placebo groups was made by the ward clinician on a simple randomization basis. This was the only person with access to the blind data and was not involved in the processing of the results. Patients and clinical staff were not allocated to the end of the study.

Group 1 consisted of patients who were receiving broad-spectrum antibiotic therapy (piperacillin with tazobactam 4.5 g administered intravenously every 6 hours [6.00 am, 12.00 am, 6.00 pm and 12.00 pm]) during endotherapy (from the onset of endoscopic treatment, for the 7 days following drainage, or 14 days in case of prolonged endoscopic drainage due to the large size of the collection). The first dose of antibiotics was administered on the day of the initial endoscopic transmural drainage procedure. In patients with renal dysfunction, the dose of antibiotics was modified depending on the renal parameters in laboratory blood tests.

Group 2 comprised patients who did not receive antibiotics during endoscopic drainage (placebo group). Patients in this group did not receive periprocedural antibiotic prophylaxis. The patients in group 2 received an equivalent volume of saline solution administered intravenously every 6 h, as mentioned above, during endotherapy (from the onset of endoscopic treatment, for the 7 days following drainage, or 14 days in case of prolonged endoscopic drainage due to the large size of the collection). The patients, study investigators, and clinical staff were blinded to the allocation until the study was completed.

### The Strategy of Interventional Treatment

In patients with symptomatic PPPFCs in the late phase of pancreatitis, transmural drainage was performed if EUS revealed that the distance between the collection wall and the gastrointestinal wall did not exceed 30 mm (Jagielski et al., 2018; Jagielski and Jackowski, 2021).

In patients with pancreatic pseudocysts, passive (without flushing through nasocystic drainage) transmural drainage was the method of choice. In case of ineffective passive transmural drainage of the pseudocyst, active transmural drainage using a nasocystic drain was performed.

In patients with WOPN, the standard endoscopic intervention method is active (with flushing through nasocystic drainage) transmural drainage. In the event that endoscopic drainage of WOPN proved ineffective, the position of the transmural nasocystic drain was changed or another fistula in a new location (multiple transluminal gateway technique [MTGT]) (Jagielski et al., 2018; Jagielski and Jackowski, 2021) was performed during the next endoscopic procedure. If the transmural drainage system did not drain the entire necrotic area or if transmural drainage was unsuccessful for WOPN patients, direct endoscopic necrosectomy was performed. Not draining area of WOPN was defined on the basis of clinical image and additional examinations. Another method of imaging of not drained area was fluoroscopic nasocystic tube-check imaging of an existing drain, where the incomplete drainage of WOPN was stated.

If endoscopic techniques with transmural access were ineffective, additional access to the collection cavity was created using percutaneous drainage (transperitoneal or retroperitoneal) or transpapillary drainage (through the major duodenal papilla).

### Endoscopic Procedures

Endoscopic procedures were performed under general anesthesia with tracheal intubation. All patients provided informed consent for the procedure. All procedures were performed by a single endoscopist with no access to the study protocol. Endoscopic procedures included carbon dioxide insufflation and the use of a linear echoendoscope (Pentax EG3870UTK, Pentax Medical, Tokyo, Japan), duodenoscope (Olympus TJF-Q180V, Olympus Corporation, Tokyo, Japan), and gastroscope (Olympus GIF-H185, Olympus Corporation). Samples of the material contained in PPPFC were collected for microbiological, cytological, and laboratory analyses.

### Endoscopic Transmural Drainage

Placement of the pancreaticogastric or pancreaticoduodenal anastomosis in the form of a transmural cystostomy was performed under EUS guidance (Jagielski et al., 2018; Jagielski and Jackowski, 2021). The anastomosis between the gastrointestinal lumen and the collection cavity was created using a 10 Fr cystotome (Cystotome CST-10, Cook Endoscopy Inc., North Carolina, USA) and dilated with a high-pressure balloon with a diameter of up to 15 mm (Cook Endoscopy or Boston Scientific). Through the stomy, a transmural metal endoprosthesis (LAMS) was inserted, measuring 16 mm in diameter and 20, 30, or 40 mm in length (Taewoong Medical or Olympus). For active transmural drainage, a 7 Fr or 8.5 Fr nasal drain (Cook Endoscopy) and 7 Fr or 8 Fr double pigtail stents (Cook Endoscopy) were inserted into the collection cavity through the LAMS. In the case of passive (without flushing through nasocystic drainage) transmural drainage, only 7 Fr or 8.5 Fr double pigtail stents (Cook Endoscopy) were used through LAMS.

### Drainage System

When active (with flushing through nasocystic drainage) transmural drainage was used, the PPPFC was flushed with saline (60 – 200 mL) through the nasal drain every 2 h during the first 48 h postoperatively and every 4–6 h on the following days.

### Treatment Efficacy Assessment

During active (with flushing through nasocystic drainage) transmural drainage, the size of the fluid collection was measured every seven days *via* abdominal ultrasound. Abdominal CECT was used to confirm complete regression of fluid collection or in cases where the patient’s clinical condition deteriorated despite ongoing treatment. Active (with flushing through nasocystic drainage) drainage was discontinued once clinical success was established, while the patients were still on passive (without flushing through nasocystic drainage) transmural drainage. After four weeks, an endoscopic procedure was performed during subsequent hospitalization, and passive transmural drainage was either continued (with transmural endoprostheses replaced) or discontinued (with the transmural endoprostheses removed). The decision to continue passive (without flushing through nasocystic drainage) transmural drainage was dependent on the fluid collection size and the presence of any disruption in the main pancreatic duct, as revealed during ERCP. If the PPPFC persisted in residual form (30 – 40 mm) or recurred (>40 mm), passive endoscopic drainage was continued and the transmural endoprostheses were replaced for another four weeks. If size of the collection was between 30 and 50 mm only the plastic “double pigtail” stents were introduced transmurally. If the size of the collection was over 50mm, the next LAMS was replaced. In cases of complete PPPFC regression, an endoscopic procedure was performed to remove the transmural endoprostheses and passive endoscopic drainage was completed.

In the case of passive (without flushing through nasocystic drainage) transmural drainage of the pancreatic pseudocyst, drainage was used during the following weeks. During the next hospitalization, an endoscopic procedure was performed and passive transmural drainage of the pancreatic pseudocyst was either continued (with transmural endoprostheses replaced according to scheme described above) or discontinued (with the transmural endoprostheses removed) depending on the size of the collection. In the case of pseudocyst regression (<30 mm), passive transmural drainage was discontinued.

After the end of endoscopic treatment the patients were placed under observation, which consisted of additional outpatient care within the surgical or gastroenterological clinic. These patients all underwent imaging control examinations of the abdomen, mostly abdominal CECT, after 3, 6, 12, and 24 months of observation or immediately in cases where patients were suspected of having clinical symptoms related to PPPFCs.

### Definitions

Technical success was defined as placement of the transmural stent with its distal flange in the PPPFC cavity and its proximal flange in the lumen of the gastrointestinal tract (stomach or duodenum) under endoscopic and radiologic guidance.

Effective transmural endoscopic drainage was considered successful if the contrast agent administered flowed freely from the PPPFC through the transmural stent without leaking out of the gastrointestinal tract or the stent. In the case of active (with flushing through nasocystic drainage) transmural drainage, the drainage was effective if the contrast agent administered through the nasal drain filled the whole cavity of the PPPFC and subsequently allowed for free outflow of content through the transmural fistula to the gastrointestinal tract.

Complications of endotherapy were defined as consequences of adverse events during endoscopic treatment.

Clinical success was defined as regression of symptoms associated with the presence of PPPFC and regression of the collection (diameter decreased to <40 mm) in imaging examinations.

Long-term success was defined as the absence of symptoms related to PPPFC and complete PPPFC regression (size decreased to <40 mm) during follow-up after endoscopic drainage.

Recurrence of PPPFC was defined as a collection size >40 mm on imaging examinations or the appearance of symptoms associated with the presence of PPPFC during follow-up.

### Statistical Analysis

All statistical calculations were conducted using STATISTICA version 12.0 (StatSoft; Tulsa, Oklahoma, United States). Quantitative variables were characterized by arithmetic means, standard deviation, minimal and maximal values (range), and 95% confidence intervals (CIs). Qualitative data were presented as numbers and percentages. To assess whether quantitative variables were normally distributed, the Shapiro-Wilk test was used. Levene’s (Brown-Forsythe) test was used to test the hypothesis of equality of variance. The significance of the differences between two groups (independent variables model) was analyzed using the Student’s t-test, Welch’s t-test (in the case of unequal variances), or Mann-Whitney U test (when Student’s t-test was not applicable or for variables measured with an ordinal scale).

The significance of differences between more than two groups was assessed using the F (ANOVA) or Kruskal-Wallis test (in case of failure to meet the applicability conditions of ANOVA). When statistically significant differences were obtained between the groups, *post hoc* tests were used (Tukey’s test for F, Dunn’s test for the Kruskal-Wallis test). In the case of the model of two related variables, the Student’s t-test or the Wilcoxon pair-order test was used (in the case of failure to meet the applicability conditions of the Student’s t-test or for variables measured on an ordinal scale). The significance of differences between more than two variables in the model of related variables was assessed by analysis of variance with repeated measures or Friedman’s test (in case of not meeting the applicability conditions of ANOVA with repeated measures or for variables measured on an ordinal scale).

The chi-squared test of independence was used for qualitative variables (with Yates’s correction for continuity when the cell number was less than 10, when Cochran’s condition was met, Fisher’s exact test).

To determine the relationship between the strength and direction of the variables, a correlation analysis was used to calculate the Pearson and/or Spearman correlation coefficients.

Statistical significance was set at P=0.05.

The analysis of the values presented in the manuscript revealed that in all applied statistical tests the power calculation was not smaller than 0.80 for significance level α=0.05.

## Results

### Patient Characteristics

The study enrolled 62 patients (12 women, 50 men; average age 49.73 [22 – 79] years) with symptomatic post-inflammatory PPFCs who underwent endoscopic transmural drainage.

Group 1 consisted of 31 patients (eight women, 23 men; mean age 51.5 [25 – 76] years) who were receiving broad-spectrum antibiotic therapy (piperacillin with tazobactam) during endotherapy.

Group 2 (placebo group) consisted of 31 patients (four women, 27 men; mean age 48.0 [22 – 79] years) without antibiotics administered during endoscopic drainage.

Detailed patients’ characteristics are presented in **Table 1**. Parameters in laboratory blood test in patients with PPPFCs before the endoscopic procedure was presented in **Table 2**.

**Table 1:** Characteristics of the patients from study group.

	Group 1 (n=31)	Group 2 (n=31)	*p*-value
**Age**, mean, [range]	51.5 [25-76]	48.0 [22-79]	0.3198
**Sex**, n, men (%)	23 (74.2%)	27 (87.1%)	0.1985
**Etiology of pancreatitis**, n, (%)			0.1797
Alcoholic	18 (58.1%)	23 (74.2%)	
Non-alcoholic	13 (41.9%)	8 (25.8%)	
**PPFCs size**, mm, mean (range)	137.3 (68-247)	156.6 (70-320)	0.2370
**Type of PPFCs**			0.6098
Pancreatic pseudocyst	15 (48.4%)	13 (41.9%)	
Walled-off pancreatic necrosis	16 (51.6%)	18 (58.1%)	
**Time from the pancreatitis to endotherapy** (days), mean (range)	78.6 (30-240)	88.0 (33-252)	0.4102
**SOFA score**, points, n (%)			0.4560
1	20 (64.5%)	16 (51.6%)	
2	11 (35.5%)	15 (48.4%)	
**CTSI** (computed tomography severity index) (points), mean (range)			0.9140
	9 (5–10)	8 (5–10)	

**Table 2:** Parameters in laboratory blood test in patients with PPPFCs before the endotherapy.

Parameter in blood test	Group 1	Group 2	*p*-value
**Leukocytes, mm^3^**	0.3542		
Mean (SD)	15.2 (6.1)	13.8 (6.3)	
Range	5.5-32.3	4.5-26.5	
**Thrombocytes, mm^3^**	0.0146		
Mean (SD)	397.4 (147.9)	314.8 (129.6)	
Range	168.0-723.0	110.0-503.0	
**C-reactive protein, mg/L**	0.2001		
Mean (SD)	142.9 (103.9)	115.7 (109.7)	
Range	1.9-404.5	0.8-344.2	
**Procalcitonin, µg/L**	0.1748		
Mean (SD)	2.1 (3.5)	3.6 (5.2)	
Range	0.0-12.5	0.0-22.5	
**Creatinine, mg/dl**	0.0001		
Mean (SD)	0.8 (0.4)	1.4 (0.6)	
Range	0.3-2.0	0.4-2.5	
**Amylase, U/L**	0.3789		
Mean (SD)	139.7 (126.0)	148.1 (106.3)	
Range	13.0-552.0	34.0-511.0	
**Bilirubin, mg/dL**	0.1881		
Mean (SD)	2.8 (4.1)	2.6 (3.2)	
Range	0.4-17.8	0.2-13.4	
**ALT, U/L**	0.7957		
Mean (SD)	313.3 (259.8)	260.4 (261.6)	
Range	36.0-849.0	49.0-1 008.0	
**AST, U/L**	0.9646		
Mean (SD)	300.1 (254.5)	268.0 (274.4)	
Range	34.0-893.0	71.0-1 107.0	

### PPPFCs Characteristics

In group 1, 16 (51.6%) patients were diagnosed with WOPN and 15 (48.4%) patients were diagnosed with pancreatic pseudocysts. In group 2, 18 (58.1%) patients were diagnosed with WOPN and 13 (41.9%) were diagnosed with pancreatic pseudocysts (p= 0.6098).

**Figure 1:**
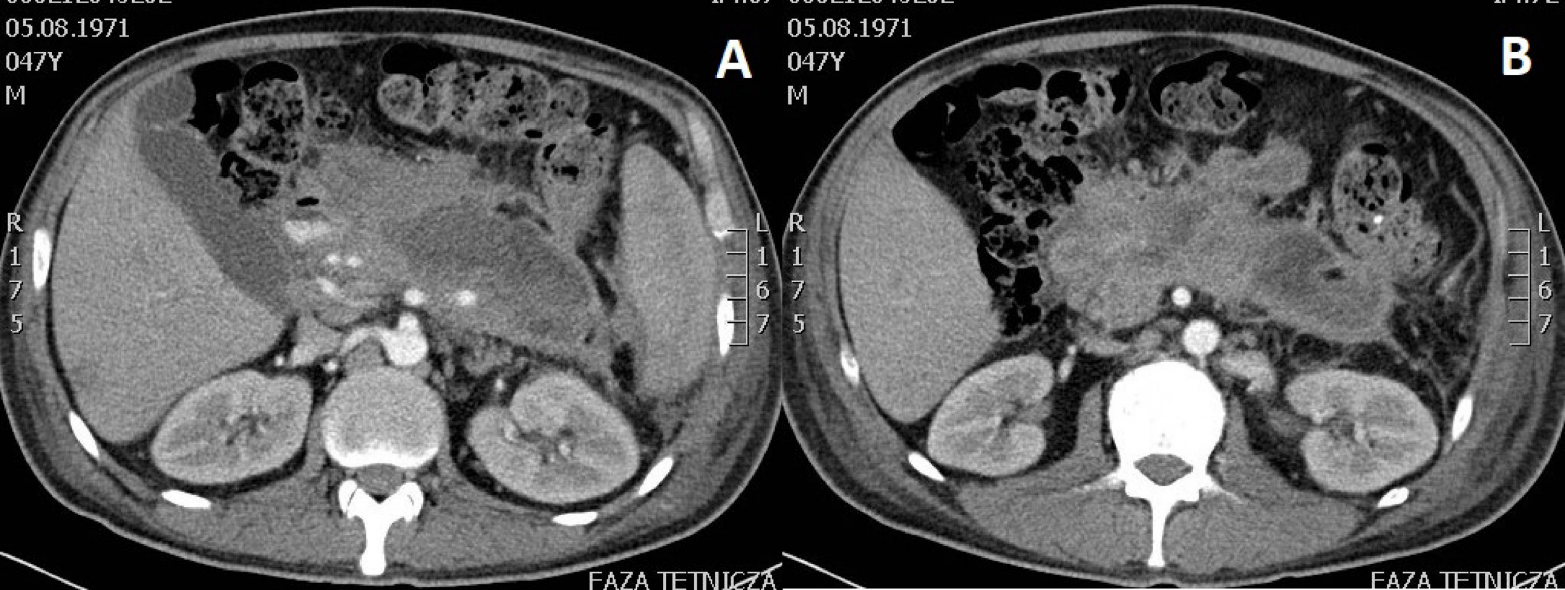
**(A, B).** Abdominal contrast-enhanced computed tomography scan performed in a patient with infected walled-off pancreatic necrosis six weeks after acute necrotic pancreatitis of alcoholic etiology. Gas bubbles seen within the lumen provide an indirect proof of infected fluid collection after excluding spontaneous fistulization of pancreatic necrosis to the gastrointestinal tract.

The average size of the PPPFC was 137.3 (68 – 247) mm in group 1 and 156.6 (70 – 320) mm in group 2 (p= 0.2370). Infections of PPPFC (**Figures 1A,B**) diagnosed on the basis of positive microbial culture content were present in 15 (48.4%) patients in group 1 and 2, respectively (p=1.0). In both groups, the most common bacterial pathogens isolated from the fluid sample were *Escherichia coli*, *Klebsiella pneumoniae*, *Enterococcus faecalis*, and *Staphylococcus epidermidis*.

The remaining indications (apart from infection with PPPFC) for endotherapy are listed in **Table 3**. In 13 (41.94%) patients in group 1 and 13 (41.94%) patients in group 2, more than one indication for endotherapy was present (p=1.0).

**Table 3:** Indications for endoscopic treatment of PPPFCs.

Indication	Group 1Number of patients, n (%)	Group 2Number of patients, n (%)	*p*-value
Infection	15 (48.4%)	15 (48.4%)	1.0
Subileus/ileus	11 (35.5%)	10 (32.3%)	0.7884
Icterus	4 (12.9%)	5 (16.1%)	0.7185
Abdominal pain	6 (19.4%)	8 (25.8%)	0.5435
Weight loss	5 (16.1%)	6 (19.4%)	0.7396

The mean time from the onset of pancreatitis to the start of endotherapy was 78.6 (30 – 240) days in group 1 and 88.0 (33 – 252) days in group 2 (p=0.4102).

Chronic pancreatitis was diagnosed in eight (25.8%) patients in group 2 and in six (19.4%) patients in group 2 (p=0.5435).

### Endoscopic Treatment Technique

**Figure 2:**
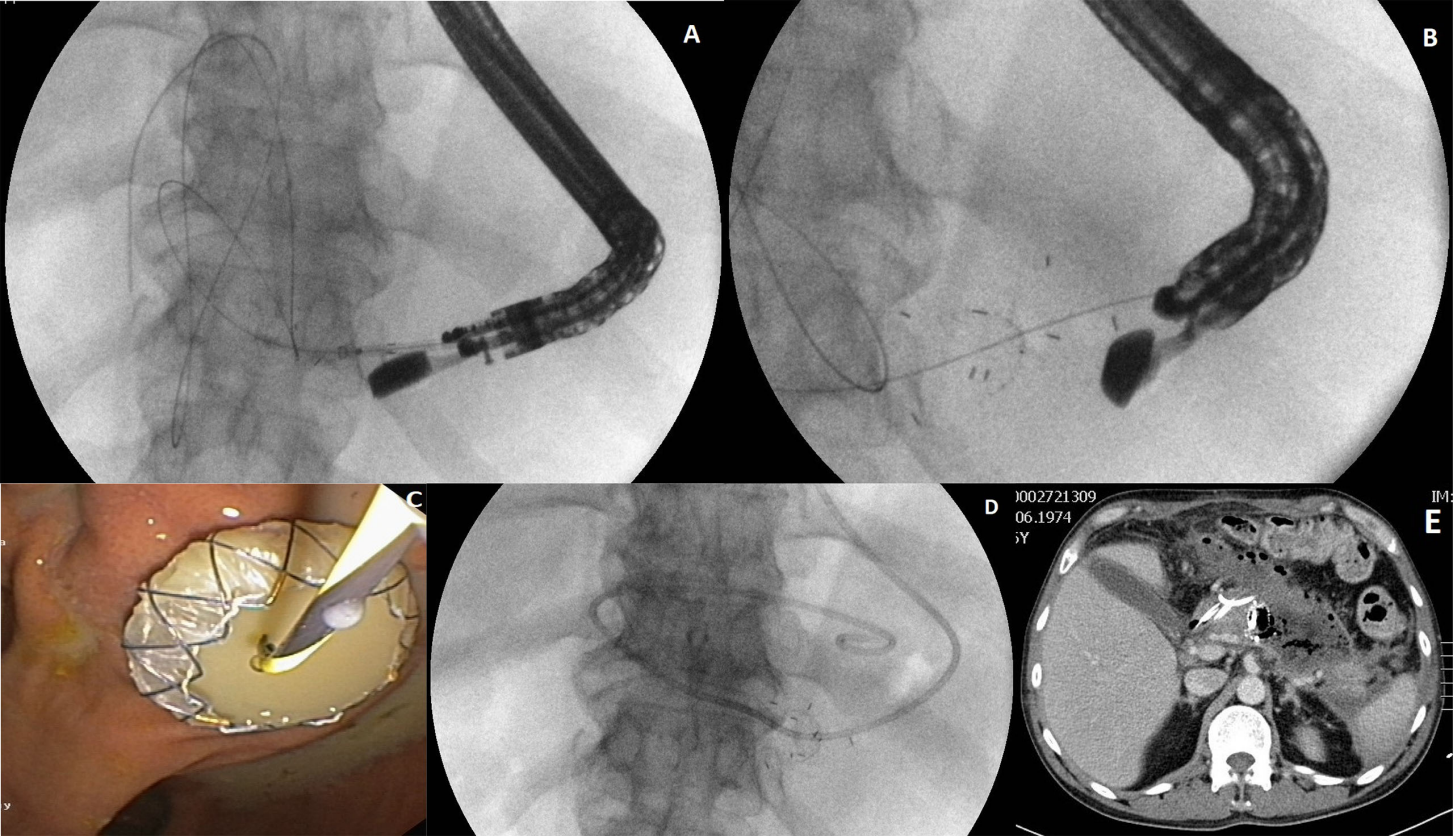
**(A–E)** Active (with flushing through nasocystic drainage) transmural drainage of a walled-off pancreatic necrosis. After the transmural fistula is created and a self-expanding stent (LAMS) is inserted transmurally **(A–C)** through the fistula, a nasal drain **(D)** is introduced along a guidewire into the necrotic area. Active transmural drainage of pancreatic necrosis is visible in computed tomography of abdomen **(E)**.

Transmural access to the PPPFCs (**Figures 2A-E**) was performed in all 62 patients (transgastric, 58; transduodenal, four). Active (with flushing through nasocystic drainage) transmural drainage was used in 16 (51.6%) patients in group 1 and in 18 (58.1%) patients in group 2 (p= 0.61). MTGT were used in four (12.9%) patients in group 1 and five (16.13%) patients in group 2 (p=0.429). Single transluminal gateway techniques were applied in the remaining 53 patients. Direct endoscopic necrosectomy was performed in 10 (32.3%) patients in group 1 and 11 (35.5%) patients in group 2 (p= 0.7884). Additional transpapillary drainage was used in three patients in group 1 and four patients in group 2. Additional percutaneous drainage was performed in four and three patients from group 1 and 2, respectively.

### Duration of Endotherapy

The mean duration of active (with flushing through nasocystic drainage) endoscopic drainage was 13 (6 – 21) days in group 1 and 14 (7 – 25) days in group 2 (P= 0.405). The average duration of passive (without flushing through nasocystic drainage) transmural drainage was 84 (29 – 265) days in group 1 and 96 (33 – 222) days in group 2 (P= 0.342). The mean number of endoscopic procedures was 3.3 (2 – 5) in group 1 and 3.4 (2 – 7) in group 2 (p=0.899).

### Complications of Endotherapy

Complications during endoscopic transmural drainage were observed in eight (25.8%) and 10 (32.3%) patients in group 1 and 2 (p=0.576), respectively. Surgical treatment of endotherapy complications was necessary in two patients from group 1 and 2, respectively. Detailed information regarding the complications is presented in **Table 4**.

**Table 4:** Complications of endoscopic treatment of patients with PPPFCs.

Complication		Group 1Number of patients, n (%)	Group 2Number of patients, n (%)	*p*-value
**Total number of complications**		8 (25.8%)	10 (32.3%)	0.576
**Upper gastrointestinal bleeding**		5 (16.13%)	6 (19.35%)	0.740
*Kind of treatment*	*Conservative*	3	4	
	*Endotherapy*	1	1	
	*Surgical*	1	1	
**Dislocation of transmural stent**		3 (9.68%)	4 (12.9%)	0.519
*Kind of treatment*	*Endotherapy*	2	3	
	*Surgical*	1	1	

The most common complication observed in both groups was gastrointestinal bleeding, it was observed in five patients in group 1 and six patients in group 2 (p= 0.740). In all cases, the cause was bleeding from the PPPFC through the transmural cystostomy into the gastrointestinal lumen. Conservative treatment with packed red blood cell transfusions and blood derivatives proved successful in seven patients with gastrointestinal bleeding during ongoing transmural drainage. Endoscopic treatment with hemostatic powder (*Hemospray*, Cook Endoscopy) sprayed into the collection cavity effectively managed bleeding in two patients. Another two patients required surgical treatment. During laparotomy, the bleeding artery, the gastroduodenal artery in one case and the splenic artery in one case, was ligated using the stick-tie technique.

### Efficacy of Endotherapy

Technical success of the transmural drainage procedure was achieved in 30 patients (96.77%) in both groups (p=1.0). In two patients, during first endoscopic procedure (performing of cystogastrostomy) inproper location of transmural stent was stated in form of proximal migration of this stent to the lumen of the collection. In both cases, the correction of the position of the LAMS with use of endoscopic forceps was performed, but there was no technical success.

Effective transmural endoscopic drainage was noted in 30 patients (96.77%) in both groups (p=1.0). In group 1, clinical success was achieved in 29 (93.55%) patients compared to 30 (96.77%) patients in group 2 (p=0.5540).

### Mortality

Mortality during endoscopic drainage was observed in two patients (one patient from group 1 and one patient from group 2) and was not associated with ongoing endoscopic treatment. All fatal cases were caused by multiple organ failure during the course of severe acute necrotizing pancreatitis in patients with pancreatic necrosis.

### Long-Term Success

During the follow-up period, which lasted an average of 598 (484 – 804) days, long-term success of endotherapy was achieved in 26 (83.9%) patients in group 1 and 24 (77.4%) in group 2 (p=0.520).

PPPFC recurrence during follow-up occurred in three (9.7%) patients in group 1 and five (16.1%) patients in group 2 (p= 0.449). All patients with recurrent PPPFC underwent successful endoscopic treatment.

### Laboratory Blood Tests

A comparison of the results of the laboratory blood tests is presented in **Table 5**-**Table 6**.

**Table 5:** Comparison of the results of C-reactive protein (mg/L) in groups of patients during endotherapy.

Day of endoscopic drainage	Group 1	Group 2	*p*-value
**1.**	0.6222		
Mean (SD)	136.7 (90.0)	125.2 (81.4)	
Range	11.5-450.7	13.3-308.7	
**3.**	0.6990		
Mean (SD)	97.4 (78.2)	83.1 (61.4)	
Range	11.2-315.1	9.4-306.7	
**5.**	0.5895		
Mean (SD)	64.2 (74.5)	47.4 (45.4)	
Range	7.9-333.1	3.0-234.5	
**7.**	0.2571		
Mean (SD)	42.6 (52.8)	27.7 (32.5)	
Range	0.9-232.0	3.4-167.6	
**10.**	0.0083		
Mean (SD)	41.1 (43.7)	19.3 (19.4)	
Range	5.7-196.5	0.9-86.1	
**15.**	0.2628		
Mean (SD)	18.6 (12.6)	16.0 (17.4)	
Range	4.5-45.2	3.3-71.5	

**Table 6:** Comparison of the results of leukocytes (mm^3^) in groups of patients during endotherapy.

Day of endoscopic drainage	Group 1	Group 2	*p*-value
**1.**	0.4592		
Mean (SD)	14.5 (5.0)	13.6 (4.7)	
Range	6.5-28.8	6.6-24.4	
**3.**	0.8360		
Mean (SD)	13.3 (5.5)	12.3 (3.2)	
Range	6.2-30.0	6.8-22.0	
**5.**	0.6309		
Mean (SD)	11.7 (4.8)	10.6 (2.5)	
Range	6.8-31.7	6.1-17.9	
**7.**	0.1797		
Mean (SD)	10.8 (4.3)	9.4 (2.1)	
Range	6.0-30.0	6.1-14.5	
**10.**	0.0791		
Mean (SD)	10.6 (3.1)	9.1 (1.8)	
Range	6.1-22.5	6.6-11.8	
**15.**	0.9158		
Mean (SD)	9.4 (1.1)	9.4 (1.6)	
Range	7.9-11.1	6.6-11.8	

## Discussion

Endoscopic treatment of PPPFCs during the course of acute pancreatitis is based on drainage of the liquid contents of the collection cavity (Loveday et al., 2008; Freeman et al., 2012; da Costa et al., 2014; Szeliga and Jackowski, 2014; Jagielski et al., 2018; Jagielski et al., 2020). Effective transmural drainage consists of free drainage of the contents of the collection *via* the transmural fistula into the gastrointestinal tract (Arvanitakis et al., 2018; Jagielski et al., 2018; Jagielski et al., 2020; Jagielski and Jackowski, 2021). No antibiotic therapy is required for successful endoscopic transmural drainage of either sterile or infected post-inflammatory PPFCs.

In the case of infected PPPFCs, free drainage of contaminated contents is accomplished by efficient active (with flushing through nasocystic drainage) transmural drainage (Jagielski et al., 2018; Jagielski et al., 2020; Jagielski and Jackowski, 2021). Thus, effective endoscopic drainage provides a means of controlling infection. No additional use of antibiotics was required for the treatment of infected PPPFCs *via* endoscopic drainage.

With regards to sterile PPPFCs, secondary contamination occurs as a result of transmural puncture for passive (without flushing through nasocystic drainage) transmural drainage (Loveday et al., 2008; Freeman et al., 2012; da Costa et al., 2014; Jagielski et al., 2018; Jagielski et al., 2020; Jagielski and Jackowski, 2021). However, efficient passive transmural drainage facilitates free outflow of the collected contents into the gastrointestinal tract (i.e., from a high-pressure compartment to a low-pressure compartment) (Loveday et al., 2008; Freeman et al., 2012; da Costa et al., 2014; Jagielski et al., 2018; Jagielski et al., 2020; Jagielski and Jackowski, 2021), thus, preventing infection. No prophylactic or periprocedural use of antibiotics is required for the treatment of primary sterile PPPFCs through efficient endoscopic drainage.

This study showed that effective endoscopic drainage of sterile post-inflammatory PPFCs requires no preventive or prophylactic use of antibiotics. In the case of contaminated PPPFC, the success of treatment depends on infection control. Antibiotic therapy is the basis of conservative treatment for infected post-inflammatory PPFCs and is responsible for controlling the infection. When endoscopic (interventional) treatment of infected PPPFCs is initiated, effective transmural drainage determines the control of infection (Jagielski et al., 2018; Jagielski et al., 2020); thus, antibiotic treatment is no longer required. No antibiotic therapy is required in cases of efficient endoscopic transmural drainage of infected post-inflammatory PPFCs.

The risk of pancreatic cystic lesions, such as post-inflammatory PPFCs, becoming infected after EUS-FNA has not been well established (Polkowski et al., 2017). The current guidelines for EUS-FNA of sterile pancreatic cystic lesions recommend the use of periprocedural antibiotic prophylaxis; however, this recommendation supported by low-quality scientific evidence (ASGE Standards of Practice Committee, 2015; Polkowski et al., 2017).

However, recently, an increasing number of published studies have emerged that undermine the validity of periprocedural antibiotic prophylaxis following EUS-FNA of pancreatic cystic lesions (Facciorusso et al., 2019; Colán-Hernández et al., 2020). A multicenter, randomized clinical trial published in 2020 showed that the risk of pancreatic cystic lesions becoming infected as a result of EUS-FNA is low and antibiotic prophylaxis is not required (Colán-Hernández et al., 2020). If periprocedural antibiotic prophylaxis is unnecessary, as the risk of infection following transmural puncture and aspiration of sterile cyst contents without endoscopic drainage is low, preventive or periprocedural use of antibiotics is even less justified in the efficient passive (without flushing through nasocystic drainage) endoscopic drainage of sterile pseudocysts and should not be used.

In relation to endoscopic drainage of post-inflammatory PPFCs, no studies supporting the use of antibiotic therapy during the course of treatment are currently available in the literature. No reference to antibiotic therapy is provided in the available guidelines for the endoscopic treatment of local complications of pancreatitis (Arvanitakis et al., 2018).

Apart from this study, the only study regarding the role of antibiotic therapy in endotherapy of PPPFCs was published in 2018 (Sahar et al., 2018). In their retrospective study, Sahar et al. compared the outcomes of endoscopic treatment of WOPN following either short-term (≤5 days) or long-term (>5 days) antibiotic prophylaxis (Sahar et al., 2018). The study showed that the outcomes of minimally invasive treatment of sterile pancreatic necrosis were comparable between the two groups (Sahar et al., 2018). However, long-term antibiotic prophylaxis has been shown to predispose patients to secondary infections, such as colitis caused by *Clostridium difficile* (Sahar et al., 2018). The authors suggested that further studies are necessary to evaluate the role and duration of antibiotic prophylaxis during drainage of P PPPFCs (Sahar et al., 2018).

Assuming that the treatment of infected PPPFCs is based on an appropriate drainage system (Loveday et al., 2008; Freeman et al., 2012; da Costa et al., 2014; Szeliga and Jackowski, 2014; Jagielski et al., 2018; Jagielski et al., 2020) to ensure infection control, the use of systemic antibiotic therapy is not justified. No systemic antibiotic therapy is required in cases of efficient drainage of infected PPPFCs formed during the course of pancreatitis, as proven in this study.

It is worth noting that in ERCP, the prophylactic use of antibiotics depends on the surgeon’s expectations of procedural success (ASGE Standards of Practice Committee, 2015). Effective endoscopic drainage of PPPFCs requires no antibiotic prophylaxis in cases of sterile collections, or antibiotic treatment in cases of infected collections. The decision to use antibiotics during endotherapy for local complications of acute and chronic pancreatitis should also be based on the anticipated effectiveness of the procedure and the efficiency of drainage in the postoperative period.

According to international guidelines, patients with acute pancreatitis should receive antibiotics in either of the following two cases: (1) extrapancreatic infection, most frequently a respiratory infection, urinary tract infection, or biliary tract infection, or (2) infection of pancreatic/peripancreatic necrotic areas, including PPPFCs (Tenner et al., 2013; Working Group IAP/APA, 2013; Arvanitakis et al., 2018; Crockett et al., 2018). PPPFCs generally become infected through translocation of the gut microbiota from the gastrointestinal tract. The most common pathogens include *Escherichia coli*, *Klebsiella pneumoniae*, *Enterococcus faecalis*, *Staphylococcus aureus*, *Pseudomonas aeruginosa*, *Proteus mirabilis*, and *Streptococcus* spp. (Olson and Allen, 1989; Stamatakos et al., 2010). Antibiotics for the treatment of infected PPPFCs may include the following agents used in monotherapy: imipenem, meropenem, piperacillin with tazobactam, or combination therapies consisting of metronidazole and one of the following antibiotics: ceftriaxone, cefotaxime, ceftazidime, and ciprofloxacin (Olson and Allen, 1989; Stamatakos et al., 2010; Villatoro et al., 2010; Tenner et al., 2013; Working Group IAP/APA, 2013; Arvanitakis et al., 2018; Crockett et al., 2018). In empirical antibiotic therapy, antibiotic agents should be selected based on good organ penetration (Tenner et al., 2013; Working Group IAP/APA, 2013; Arvanitakis et al., 2018; Crockett et al., 2018). In the case of targeted antibiotic therapy, the antibiotic agent should be selected mainly based on swab culture/antibiogram results (Olson and Allen, 1989; Stamatakos et al., 2010; Villatoro et al., 2010; Tenner et al., 2013; Working Group IAP/APA, 2013; Arvanitakis et al., 2018; Crockett et al., 2018).

However, it should be stressed that the above recommendations concerning antibiotic therapy in acute pancreatitis only apply to patients receiving conservative rather than interventional treatment (Tenner et al., 2013; Working Group IAP/APA, 2013; Arvanitakis et al., 2018; Crockett et al., 2018). No recommendations regarding antibiotic therapy are available for patients with acute pancreatitis undergoing interventional procedures (Arvanitakis et al., 2018).

Irrespective of the technique used in minimally invasive treatment of the sequelae of acute pancreatitis, the essence of interventional treatment consists of drainage of the liquid contents (Loveday et al., 2008; Freeman et al., 2012; da Costa et al., 2014; Szeliga and Jackowski, 2014; Jagielski et al., 2018; Jagielski et al., 2020). Efficient drainage facilitates the free evacuation of liquid content from the collection cavity (Arvanitakis et al., 2018; Jagielski et al., 2018; Jagielski et al., 2020; Jagielski and Jackowski, 2021). The establishment of an appropriate drainage system is the basis for the effective treatment of pancreatitis complications, such as PPPFCs (Loveday et al., 2008; Freeman et al., 2012; da Costa et al., 2014; Szeliga and Jackowski, 2014; Jagielski et al., 2018; Jagielski et al., 2020). Thus, effective drainage prevents proliferation of microorganisms by continuously draining the contents from the collection cavity during treatment; thus, antibiotic prophylaxis and antibiotic treatment are not required for sterile and infected collections, respectively. Effective transmural drainage of PPPFCs does not require antibiotic therapy, and thus should contribute to reducing the abuse of antibiotics.

An abscess is an inflammatory collection formed during an infection that is filled with purulent material (Sartelli et al., 2017; Perrone et al., 2020). For years, drainage has been known to be an effective method of abscess treatment (Sartelli et al., 2017; Perrone et al., 2020). An abdominal abscess is a collection of cellular debris, enzymes, and liquefied remains from an infectious or noninfectious source (Sartelli et al., 2017; Perrone et al., 2020). A separate subgroup of abdominal abscesses consists of retroperitoneal abscesses (Sartelli et al., 2017; Perrone et al., 2020), including contaminated PPPFCs localized in this region resulting from pancreatitis, that is, infected pancreatic pseudocysts and WOPN. Abdominal sepsis control is the recommended treatment for abdominal fluid collection (abscesses), including post-inflammatory peritoneal fluid collection (Sartelli et al., 2017; Perrone et al., 2020). Indeed, antibiotic therapy is recommended and even necessary to control infection until interventional treatment of post-inflammatory PPFCs is initiated (Tenner et al., 2013; Working Group IAP/APA, 2013; Arvanitakis et al., 2018; Crockett et al., 2018). However, when interventional treatment is initiated, that is, effective drainage is accomplished using endoscopic techniques, the infection is controlled by effective transmural drainage; therefore, ongoing antibiotic treatment should not be continued. In the case of infected PPPFCs, interventional management is based on the evacuation of infected necrotic content inside and outside the pancreas, which is key to the successful treatment of the sequelae of acute pancreatitis and the control of infection (Loveday et al., 2008; Freeman et al., 2012; da Costa et al., 2014; Szeliga and Jackowski, 2014; Jagielski et al., 2018; Jagielski et al., 2020).

As stated above, the reduction in antibiotic use in daily clinical practice is consistent with the principles of rational antibiotic therapy. Abuse of antibiotics leads to increased costs of medical procedures and possible allergic reactions to drugs; however, it also increases the risk of drug resistance against different types of microorganisms. Irrational antibiotic therapy, understood as the excessive and unjustified use of antibiotics, can lead to secondary infections, such as colitis caused by *Clostridium difficile* or fungal infections.

This study is the first randomized trial to prove that the effective endoscopic drainage of sterile PPPFCs requires no preventive or prophylactic use of antibiotics. In infected PPPFCs, antibiotic therapy is not required for effective endoscopic transmural drainage.

The main limitation of our study is that it was conducted on a selected group of patients from one medical center. Future, multi-center studies with larger sample sizes are required to validate our results.

In summary, the effective endoscopic drainage of post-inflammatory PPFCs reduces the use of antibiotics in everyday clinical practice, which is consistent with the principles of rational antibiotic therapy.

## Data Availability Statement

The raw data supporting the conclusions of this article will be made available by the authors, without undue reservation.

## Ethics Statement

The studies involving human participants were reviewed and approved by Ethics Committee at the Collegium Medicum of Nicolaus Copernicus University (Approval Number KB 294/2020). The patients/participants provided their written informed consent to participate in this study.

## Author Contributions

Conceptualization was performed by MatJ, WK, JP, and MarJ. Formal analysis was performed by MatJ, WK, JP, and MarJ. The methodology was performed by MatJ, WK. Project administration was performed by WK, JP, and MarJ. Endoscopic procedures was performed by MatJ. The writing of the original draft was performed by MatJ, WK and JP. Editing was performed by MatJ, and MarJ. All authors (MatJ, WK, JP, and MarJ) revised and approved the submitted version of the manuscript.

## Conflict of Interest

The authors declare that the research was conducted in the absence of any commercial or financial relationships that could be construed as a potential conflict of interest.

## Publisher’s Note

All claims expressed in this article are solely those of the authors and do not necessarily represent those of their affiliated organizations, or those of the publisher, the editors and the reviewers. Any product that may be evaluated in this article, or claim that may be made by its manufacturer, is not guaranteed or endorsed by the publisher.
